# Supplementary material for: Needs assessment for creation of a platform trial network in metabolic-dysfunction associated steatohepatitis
Source: Commun Med (Lond). 2024 Jul 16;4:144. doi: 10.1038/s43856-024-00560-5 (PMC11253004; doi:10.1038/s43856-024-00560-5)

Supplementary Information File.

List of affiliations in the EU-PEARL consortium.

List of MASH EU-PEARL investigators:

Core group: Nicholas A. DiProspero[1], Vlad Ratziu[2], Juan M. Pericàs[3], Mette Skalshøj Kjær[4], Quentin M. Anstee[5,6], Frank Tacke[7], Peter Mesenbrinck[8], Jesús Rivera-Esteban[3], Franz Koenig[9], Elena Sena[3], Sergio Muñoz[3], Joan Genescà[3], Raluca Pais[2], Leila Kara[2], Elias Meyer[9], Anna Duca[1], Timothy Kline[1], Anders Aaes-Jørgensen[4], Tania Balthaus[10], Natalie de Preville[10], Lingjiao Zhang[1], George Capuano[1], Salvatore Morello[1], Tobias Mielke[1], Sabina Hernandez Penna[8], Martin Posch[9].

Affiliations:

1. Janssen Research and Development, Raritan, New Jersey, USA. **2**. Department of Hepatology, Pitié-Salpetriere Hospital, University Paris 6, Paris, France. **3**. Liver Unit, Vall d'Hebron University Hospital, Vall d'Hebron Institute for Research (VHIR), Spanish Network of Biomedical Research on Digestive and Liver Diseases (CIBERehd), Barcelona, Spain **4.** Novo Nordisk A/S, Bagsvaerd, Denmark **5.** Liver Unit, The Newcastle upon Tyne Hospitals NHS Foundation Trust, Newcastle upon Tyne, UK. **6.** Translational and Clinical Research Institute, Newcastle University, Newcastle upon Tyne, UK. **7.** Department of Hepatology and Gastroenterology, Charité - Universitätsmedizin Berlin, Berlin, Germany. **8.** Novartis Pharmaceuticals Corporation, One Health Plaza, East Hanover, NJ, USA **9.** Center for Medical Data Science, Medical University of Vienna, Vienna, Austria. **10.** Translational and Clinical Research, Metabolism Innovation Pole, Institut de Recherches Internationales Servier, Suresnes Cedex, France

Survey participants/collaborating investigators: Markus Peck-Radosavljevic[11], Anja Geerts[12,13], Lotte Gluud[14,15], Maja Thiele[16], Hannele Yki-Järvinen[17,18], Jérôme Boursier[19,20], Christian Trautwein[21,22], Philipp Lutz[23,24], Johannes Wiegand[25], Jörn Schattenberg[26,27], Andreas Geier[28], George Papatheodoridis[29], Giulio Marchesini[30], Luca Valenti[31,32], Fabio Nascimbeni[33], Salvatore Petta[34], Patrizia Burra[35], Luca Miele[36,37], Ger Koek[38], Robert de Knegt[39], Helena Cortez-Pinto[40], Sofia Carvalhana[41], Antonio Olveira[42], Teresa Broquetas[43,44], Germán Soriano[45-47], Jesús Bañales[48], Rosa Martín-Mateos[49], Juan Turnes[50], Manuel Romero-Gomez[51], Salvador Benlloch[52], Mattias Ekstedt[53], Hannes Hagström[54,55], Jean-François Dufour[56], Lynsey Corless[57], Richard Parker[58], Emmanuel A Tsochatzis[59], David Sheridan[58], Christopher D Byrne[60], Münnevver Demir[61], Mercè Vergara[62], Vanesa Bernal[63], Conrado Fernández-Rodríguez[64], Carmelo García-Monzón[65], Rafael Bañares[66-68], Raul Andrade[69], Henning Grønbæk[70,71], Elisabetta Bugianesi[72], Lucia Brodosi[73,74], Alessio Aghemo[75,76], Alessandra Mangi[illegible]

Affiliations: **11.** Department of Internal Medicine and Gastroenterology (IMuG), Hepatology, Endocrinology, Rheumatology and Nephrology Including Emergency Medicine (ZAE), Klinikum Klagenfurt am Wörthersee, 9020 Klagenfurt, Austria. **12.** Department of Internal Medicine and

Pediatrics, Hepatology Research Unit, Ghent University, Ghent, Belgium. **13.** Liver Research Center Ghent, Ghent University, Ghent University Hospital, Ghent, Belgium. **14.** Department of Clinical Medicine, Faculty of Health and Medical Sciences, University of Copenhagen, Copenhagen, Denmark. **15.** Gastro Unit, Medical Division, Copenhagen University Hospital - Amager and Hvidovre Hospital, Hvidovre, Denmark.
16. Center for Liver Research, Odense University Hospital, University of Southern Denmark, Odense, Denmark. **17**. Department of Medicine, University of Helsinki and Helsinki University Hospital, Helsinki, Finland. **18**. Minerva Foundation Institute for Medical Research, Helsinki, Finland. **19**. Hepato-Gastroenterology Department, University Hospital, Angers, France. **20**. HIFIH Laboratory, UPRES 3859, SFR 4208, LUNAM University, Angers, France. **21**. Gastroenterology, Hepatology and infectious Diseases Department, University Hospital RWTH Aachen, Aachen, Germany. **22**. Site: Aachen, Center for Integrated Oncology Aachen, Bonn, Cologne and Duesseldorf (CIO ABCD), Aachen, Germany. **23.** Department of Internal Medicine I, University Hospital of Bonn, Bonn, Germany. **24.** Department of Internal Medicine I, University Hospital of Bonn, Bonn, Germany. **25.** Division of Hepatology, Department of Medicine II, Leipzig University Medical Centre, Leipzig, Germany. **26.** Metabolic Liver Disease Research Program, I. Department of Medicine, University Medical Center of the Johannes Gutenberg-University, Mainz, Germany. **27.** Department of Internal Medicine II, Saarland University Medical Center, Homburg, Germany. **28.** Department of Medicine II, Division of Hepatology, University Hospital Wurzburg, Würzburg, Germany **29.** Academic Department of Gastroenterology, Medical School of National and Kapodistrian University of Athens, Athens, Greece. **30.** Universita' degli Studi di Bologna, Bologna, Italy. **31.** Precision Medicine Lab, Biological Resource Center and Department of Transfusion Medicine, Fondazione IRCCS Ca' Granda Ospedale Maggiore Policlinico Milano, Via F Sforza 35, 20122, Milan, Italy. **32.** Department of Pathophysiology and Transplantation, Università degli Studi di Milano, Milan, Italy. **33.** Internal and Metabolic Medicine, Department of Medical and Surgical Sciences for Children & Adults, AOU di Modena, University of Modena and Reggio Emilia, 41126 Modena, Italy. **34.** Sezione di Gastroenterologia, PROMISE, University of Palermo, Palermo, Italy. **35.** Multivisceral Transplant Unit, Department of Surgery, Oncology and Gastroenterology, Padua University Hospital, Padua, Italy. **36.** Department of Medical and Surgical Sciences, CEMAD, Fondazione Policlinico Universitario Agostino Gemelli IRCCS, Rome, Italy. **37.** Department of Translational Medicine and Surgery, Catholic University of Sacred Heart, Rome, Italy. **38.** Department of Internal Medicine, Division of Gastroenterology/Hepatology, Maastricht UMC+, Maastricht, Netherlands. **39.** Department of Gastroenterology and Hepatology, Erasmus MC University Medical Center, Rotterdam, The Netherlands. **40.** Clínica Universitária de Gastrenterologia, Faculdade de Medicina, Universidade de Lisboa, Portugal. **41.** Departamento de Gastrenterologia, Centro Hospitalar Universitário Lisboa Norte, Lisbon, Portugal. **42.** Hospital Universitario La Paz, Madrid, Spain. **43.** Liver Section, Gastroenterology Department, Hospital del

Mar, Barcelona 08003, Spain. **44.** Institut Hospital del Mar D'Investigacions Mèdiques, PSMAR, Barcelona 08003, Spain. **45.** Department of Gastroenterology, Hospital Santa Creu i Sant Pau, Institut de Recerca Sant Pau, Barcelona, Spain. **46.** Department of Medicine, Universitat Autònoma de Barcelona, Barcelona, Spain. **47.** Centro de Investigación Biomédica en Red de Enfermedades Hepáticas y Digestivas (CIBERehd), Instituto de Salud Carlos III, Madrid, Spain. **48.** Department of Liver and Gastrointestinal Diseases, Biodonostia Health Research Institute-Donostia University Hospital, University of the Basque Country (UPV/EHU), San Sebastian, Spain. Spanish Network of Biomedical Research on Digestive and Liver Diseases (CIBERehd), 10IKERBASQUE, Basque Foundation for Science, Bilbao, Spain **49.** Servicio de Gastroenterología y Hepatología, Hospital Universitario Ramón y Cajal, Madrid. Universidad de Alcalá, Madrid, España; Instituto Ramón y Cajal de Investigación Sanitaria (IRYCIS), Madrid, España; Centro de Investigación Biomédica en Red de Enfermedades Hepáticas y Digestivas (CIBEREHD), Instituto Salud Carlos III, Madrid, España. **50.** Department of Gastroenterology and Hepatology, Pontevedra University Hospital Complex, IIS Galicia Sur, Pontevedra, Spain; Health Research Institute (IIS) Galicia Sur, Pontevedra, Spain. **51.** Enfermedades Digestivas y Ciberehd, Hospital Universitario Virgen del Rocío, Instituto de Biomedicina de Sevilla (CSIC/HUVR/US), Universidad de Sevilla, Sevilla, España. **52.** Servicio de Digestivo, Hospital Arnau de Vilanova, Universidad CEU-Cardenal Herrera, Valencia, CIBERhed-Instituto de salud Carlos III, Madrid, Spain **53.** Division of Diagnostics and Specialist Medicine, Department of Health, Medicine and Caring Sciences, Linköping University, Linköping, Sweden. **54.** Unit of Gastroenterology and Rheumatology, Department of Medicine Huddinge, Karolinska Institutet, Stockholm, Sweden. **55.** Department of Internal Medicine, Section of Gastroenterology, Södersjukhuset, Stockholm, Sweden. **56.** Center for Digestive Diseases Lausanne, Lausanne, Switzerland. **57.** Department of Gastroenterology, Hepatology and Endoscopy, Hull University Teaching Hospitals NHS Trust, Hull, UK; Hull York Medical School, Hull, UK. **58.** Institute of Translational and Stratified Medicine, University of Plymouth, Plymouth, UK. **59.** UCL Institute for Liver and Digestive Health, Royal Free Hospital and UCL, London, UK. **60.** Nutrition and Metabolism, Faculty of Medicine, University of Southampton, Southampton, UK; National Institute for Health and Care Research Southampton Biomedical Research Centre, University Hospital Southampton, Southampton, UK **61.** Department of Hepatology and Gastroenterology, Charité University Medicine, Campus Virchow Clinic and Campus Charité Mitte, Charite Universitatsmedizin Berlin, Berlin, Germany. **62.** Centro de Investigación Biomédica en Red de Enfermedades Hepáticas y Digestivas (CIBERehd) Instituto de Salud Carlos III, España; Unidad de Hepatología, Servicio Digestivo, Hospital Universitari Parc Taulí, Institut d'Investigació i Innovació Parc Taulí I3PT, Universitat Autònoma de Barcelona, Sabadell, España; Grupo de trabajo sobre «Hígado graso no alcohólico» de la Societat Catalana de Digestologia, Barcelona, España. **63.** Hospital Universitario Miguel Servet, Zaragoza, Spain. **64.** Hospital Universitario Fundación de Alcorcón, Universidad Rey Juan Carlos, Móstoles, Spain. **65.** Liver Research Unit, Hospital Universitario Santa Cristina Instituto

de Investigación Sanitaria Princesa Madrid, Madrid, Spain. **66.** Servicio de Aparato Digestivo, Hospital General Universitario Gregorio Marañón, Madrid, Spain. **67.** Instituto de Investigación Sanitaria Gregorio Marañón, Madrid, Spain. **68.** Centro de Investigación Biomédica en Red de Enfermedades Hepáticas y Digestivas, Madrid, Spain. **69.** Servicios de Aparato Digestivo y Farmacología Clínica, Hospital Universitario Virgen de la Victoria, Instituto de Investigación Biomédica de Málaga y Plataforma en Nanomedicina-IBIMA Plataforma BIONAND, Universidad de Málaga, Málaga, Spain; Centro de Investigación Biomédica en Red Enfermedades Hepáticas y Digestivas (CIBERehd), Madrid, Spain; Plataforma de Investigación Clínica y Ensayos Clínicos UICEC-IBIMA, Plataforma ISCIII de Investigación Clínica, Madrid, Spain. **70.** Department of Hepatology and Gastroenterology, Aarhus University Hospital, Aarhus, Denmark. **71.** Department of Clinical Medicine, Aarhus University, Aarhus, Denmark. **72.** Department of Medical Sciences, Division of Gastroenterology, University of Turin, Turin, Italy. **73.** Department of Medical and Surgical Sciences, University of Bologna, Bologna, Italy. **74.** Clinical Nutrition and Metabolism Unit, IRCCS-Azienda Ospedaliero-Universitaria Di Bologna, Bologna, Italy. **75.** Division of Internal Medicine and Hepatology, Department of Gastroenterology, IRCCS Humanitas Research Hospital, Rozzano, Italy. **76.** Department of Biomedical Sciences, Humanitas University, Pieve Emanuele, Italy. **77.** Liver Unit, Department of Medical Sciences, Fondazione "Casa Sollievo della Sofferenza" IRCCS, 71013 San Giovanni Rotondo, Italy. **78.** Department of Specialistic Medicines, Gastroenterology Unit, University of Modena & Reggio Emilia, University Hospital of Modena and Reggio Emilia, Modena, Italy. **79.** Liver Unit-CHTMAD, Vila Real, Portugal.

Supplementary Note 1: Reach out survey for the NASH IRP CRN.

NASH IRP: Assessment of potential interest and capacity for the set-up of a platform trial

Thank you for your time in answering this survey. Please, make sure you read the Supplementary information materials before answering to be familiar with the concepts discussed below. This is a non-binding survey. It will be used to assess potential candidates' interests, concerns, and input to build a clinical research network to conduct a Phase 2b NASH platform trial in the near future. It is not required to answer all the questions, but we appreciate any information or opinions that might be relevant to set-up a NASH platform trial or adaptations to its master protocol.

Should you have any additional concerns or suggestions, please reach out to Elena Sena (elena.sena@vhir.org)

The Fundació Hospital Universitari Vall d'Hebrón - Institut de Recerca (VHIR) is an academic hospital and research centre, which is responsible for the processing of your personal data. The purpose of the processing is to be able to manage your request for services to the Unidad de Alta Tecnologia/High Technology Unit (UAT) or Unidad de Estadística y Bioinformática/Statistics and Bioinformatics Unit (UEB). You may exercise your rights of access, rectification, deletion, opposition, portability and limitation by sending an e-mail to lopd@vhir.org or to the VHIR Data Protection Officer at dpd@ticsalutsocial.cat. For more information about VHIR's privacy policy, please visit the Personal Data Protection Website

1. Investigator and site contact details

1.1 Name

1.2 Institution

1.3 Type of site:

University Hospital ☐ Non-teaching hospital ☐ Private Clinic ☐

Research Institute ☐ Clinical trial unit/CRO ☐ Other (specify your site type) ☐

1.4 E-mail address

1.5 Do you participate in any national or international NAFLD/NASH registry?

Yes☐ No☐

(Please, specify)

1.6 Is your center participating in the LITMUS project (European Commission-EPFIA IMI2 grant agreement No. 777377)?

Yes☐ No☐

1.7 Is your center a European University Hospital Alliance (EUHA) member?

Yes☐ No☐

2. Current landscape of NASH clinical trials

2.1 Currently, what do you think is the NASH population with a more significant therapeutic unmet need?

NASH without fibrosis or at early stages of fibrosis☐

NASH with significant fibrosis☐

NASH with advanced fibrosis☐

NASH cirrhosis☐

2.2 How difficult would you say it is to identify candidates for NASH trials?

No or slightly difficult☐

Moderately☐

Very difficult☐

Extremely difficult☐

2.3 What would you say is the main barrier for investigators in identifying potential candidates?

2.4 What is the main barrier for patients to be willing or able to participate in a NASH trial?

2.5 Do you envision regulatory approval of NASH drugs not based on histology findings?

Yes☐ No☐

2.6 Do you think that NASH trials could be designed and run more efficiently, e.g., regarding trial duration and the chances of participants receiving a therapeutically active compound instead of placebo/standard of care?

Yes☐ No☐

2.7 What type of stakeholder is more likely to take the initiative to change the way NASH trials are conducted within the next 5-10 years?

Academic institutions☐

Drug industry☐

Academic institutions following health authorities' initiative either at the national or European level☐

Industry and patients' associations☐

Industry and academic institutions☐

3. Investigator and site' experience on NASH clinical trials

3.1 How many NASH clinical trials are you currently conducting in your department?

3.2 Which phase are the clinical trials you are currently participating in?

Phase I ☐ Phase IIa☐ Phase IIb☐ Phase III☐

3.3 Has your site a Clinical Trials/Research Unit (CTU)?

Yes☐ No☐

3.4 How many years of experience do you have conducting clinical research?

3.5 On average, how many patients with NASH do you see per year?

3.6 Do you routinely use non-invasive tests (NITs) to diagnose and monitor NASH in clinical trials or clinical practice?

Yes☐ No☐

3.7 If yes, which ones?

MRI☐ Serological tests☐ Transient elastography☐ Other (please, specify)☐

3.8 How many liver biopsies for diagnosing suspected NASH do you perform per month?

3.9 Does your site/team have the following human resources and expertise?

Clinical sub-investigator Yes☐ No☐

Study coordinator Yes☐ No☐

Study nurse Yes☐ No☐

Pharmacist Yes☐ No☐

Laboratory technician Yes☐ No☐

Pathologist Yes☐ No☐

MRI-PDFF Yes☐ No☐

Fibroscan® Yes☐ No☐

4. Building an Integrated Research Platform to conduct a NASH Platform trial

4.1 Are you familiar with adaptive trial designs?

Yes☐ No☐

4.2 Have you ever participated in a platform trial?

Yes☐ No☐

4.3 Do you think that the specific characteristics of the NASH landscape (e.g., the prevalence of the disease, diagnostic criteria, lack of approved drugs, etc.) make NASH a good candidate in terms of necessity, feasibility, and interest from funders and regulators to set up a platform trial?

Yes☐ No☐ (Please, specify)

4.4 Are you comfortable participating in a clinical trial that allows interim decisions to stop an arm due to futility/safety reasons?

Yes☐ No☐ (Please, specify)

4.5 Do you believe that a platform trial can provide distinct benefits to NASH patients?

No benefits ☐ Few benefits ☐ Moderate benefits ☐ Many benefits☐

4.6 Would patients understand the additional benefit of participating in a platform trial?

Yes☐ No☐ (Please, specify)

4.7 If patients at your site could be both eligible for the platform trial and other concurrent sponsored standalone clinical trials on NASH, do you see any issue regarding feasibility or recruitment ability?

Yes☐ No☐ (Please, specify)

4.8 Please, arrange the following items as of their relevance for you to participate in a NASH PT:

More chances for patients to receive an active compound ☐

Scientific endeavor, innovation potential ☐

Possibility to participate in governance bodies of the IRP (e.g., scientific committee)

Scientific publications ☐

Economic compensation at least as good as in a traditional standalone industry-funded CT☐

4.9 Please, add any other element that you consider to be important either to participate in the PT or that might dissuade you from doing so:

4.10 Which of the following organizations would you prefer as the sponsor for the NASH Integrated Research Platform (IRP)?

Academic institution☐ Non-profit organization☐

4.11 Do you think the NASH IRP can stand alone with an academic CRO or it needs a global CRO specialized in NASH?

Academic CRO☐ NASH specialized CRO☐

4.12 Is it better to establish the NASH IRP only in Europe or it should be a global platform trial including sites from other geographical locations?

Europe☐ Global☐

4.13 Would you agree with a progressive geographical deployment of the clinical network that runs along the number of investigational medical product (IMP) tested and the funds available?

Yes☐ No☐

4.14 Are you be more comfortable working with IMP from large companies or small biotech for a future NASH IRP?

Large companies☐ Small biotech☐

4.15 Would you be open to master protocol modifications that replace liver biopsy by non-invasive biomarkers as the primary endpoint?

Yes☐ No☐

4.16 Would you rather adapt the current master protocol to a Phase 2a trial that might allow graduating IMP to Phase 2b/3?

Yes☐ No☐ (Please, specify)

4.17 The master protocol includes storing and digitalizing liver biopsy slides so each IMP owner can decide whether to use artificial intelligence techniques to enhance cross-pathologist readings. Do you agree with that?

Yes☐ No☐ (Please, specify why)

4.18 Cirrhotic patients were not included in the Master protocol. Do you think that future master protocol models should be contemplated to include this population?

Yes☐ No☐ (Please, specify)

5. Open questions about interest

5.1 Would you be interested in participating the platform trial?

Yes ☐ Only if funded by the industry from the beginning ☐

No ☐ (Could you indicate why?)

5.2 If there was a public call to fund the NASH IRP, would you be keen on participating in a consortium aimed at establishing the NASH IRP?

Yes ☐ No ☐

5.2.1 Are you interested in being contacted in the future about the progression of the IRP for NASH?

Yes ☐ No ☐

5.3 Do you give permission to publish the information extracted from this survey in a scientific article?

Yes ☐ No ☐

5.3.1 If YES, would you like your name included in the list of authors (either individual or collective authorship)?

Yes ☐ No ☐

5.4 Could you recommend 2-3 colleagues that might be interested in being part of a platform trial?

Supplementary Figure 1: Flowchart depicting the steps and timeline for the survey

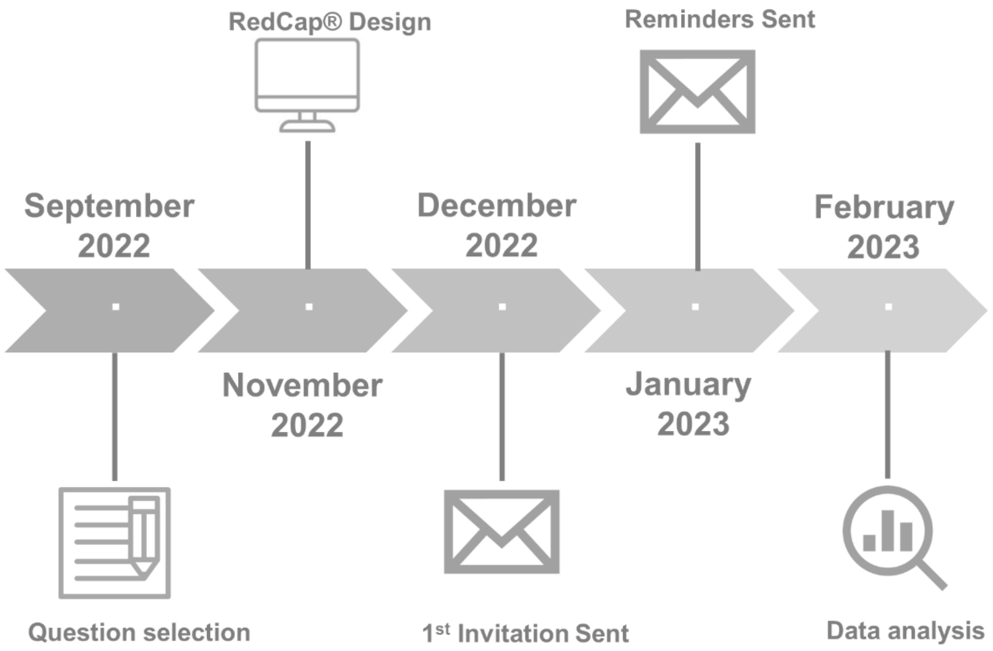

Supplement: Supplementary file 2 — Supplementary Information [file 43856_2024_560_MOESM2_ESM.pdf]
