# Supplementary material for: Needs assessment for creation of a platform trial network in metabolic-dysfunction associated steatohepatitis
Source: Commun Med (Lond). 2024 Jul 16;4:144. doi: 10.1038/s43856-024-00560-5 (PMC11253004; doi:10.1038/s43856-024-00560-5)
Supplement: Supplementary file 3 — Description of Additional Supplementary Files [file 43856_2024_560_MOESM3_ESM.pdf]

Description of Additional Supplementary Files

File name: Supplementary Data 1

File Description: List of preselected sites to be invited to the survey.

File name: Supplementary Data 2

File Description: Summary of the experience of invited sites in MASH clinical trials

File name: Supplementary Data 3

File Description: Source data for Figure 3
